# In vitro cytotoxic mechanisms of Pt(O,O′-acac)(γ-acac)(DMS): mitochondrial dysfunction and impaired autophagy in U251 cell line

**DOI:** 10.1038/s41420-025-02918-7

**Published:** 2026-01-09

**Authors:** L. Gaiaschi, F. De Luca, C. R. Girelli, G. Milanesi, E. Roda, F. P. Fanizzi, M. Grimaldi, M. G. Bottone

**Affiliations:** 1 Laboratory of Cell Biology and Neurobiology, Department of Biology and Biotechnology “L. Spallanzani”, University of Pavia, Pavia, Italy; 2 Department of Biological and Eviromental Sciences and Technologies (DISTeBA), University of Salento, Lecce, Italy; 3 ICS Maugeri SpA, IRCCS Pavia, Laboratory of Clinical & Experimental Toxicology, Pavia Poison Centre, National Toxicology Information Centre, Toxicology Unit, Pavia, Italy

**Keywords:** Chemotherapy, CNS cancer

## Abstract

Glioblastoma stands as the deadliest primary brain malignancy in adults, primarily due to its resistance to conventional treatments and the restrictive nature of the blood–brain barrier (BBB). Cisplatin (CDDP), a widely used chemotherapeutic, demonstrates limited efficacy against glioblastoma owing to systemic toxicity and insufficient BBB penetration. To overcome these hurdles, we tested the platinum(II) complex [Pt(O,O′-acac)(γ-acac)(DMS)], indicated as Pt(acac)₂(DMS), known for its improved lipophilicity, ability to disrupt mitochondrial function, and reduced neurotoxic profile. Compared to CDDP, Pt(acac)₂(DMS) induced a targeted and prolonged cytotoxic response in U251 glioblastoma cells, promoting mitochondrial dysfunction, cell cycle arrest, and modulation of autophagy, while sparing primary human astrocytes. Our findings indicate that Pt(acac)₂(DMS) may overcome key limitations of cisplatin, including toxicity issues and resistance associated with autophagic adaptation, highlighting its promise as a potential therapeutic candidate for glioblastoma treatment.

## Introduction

Malignant gliomas are the most aggressive type of primary brain tumors in adults, with glioblastoma (GB, WHO grade IV) being the most common and deadly form. Despite advances in molecular characterization and treatment approaches, the prognosis for GB remains poor, with median survival rarely exceeding 15 months under standard therapy (surgical resection followed by radiotherapy and temozolomide) [1]. Treatment failure is largely due to GB’s strong resistance to apoptosis, its high intratumoral heterogeneity, and the presence of the blood–brain barrier (BBB), which limits drug delivery to the tumor [2].

Cisplatin (CDDP), a widely used platinum-based chemotherapy, is effective in treating several solid tumors, including ovarian, testicular, and lung cancers. Its primary mechanism of action involves binding to DNA, thereby blocking replication and transcription. CDDP also contributes to oxidative stress and disrupts intracellular calcium (Ca²⁺) balance, altering mitochondrial functions [3]. However, the clinical use of CDDP is limited by serious side effects, including kidney, nerve, and hearing damage, as well as the development of drug resistance. Resistance mechanisms include enhanced DNA repair, increased detoxification via glutathione, reduced drug uptake, and overexpression of anti-apoptotic proteins [4].

To overcome these challenges in glioblastoma treatment, new therapeutic approaches have been studied [5, 6], and platinum-based compounds have been designed [7]. One promising candidate is the complex [Pt(O,O′-acac)(γ-acac)(DMS)], here indicated as Pt(acac)₂(DMS), which contains acetylacetonate and dimethylsulfide ligands. Preclinical studies in multiple tumor xenograft models, including breast cancer and mesothelioma, have demonstrated potent antitumor efficacy and a favorable safety profile in vivo [8, 9]. Moreover, pharmacokinetic evaluations have confirmed detectable platinum levels in brain tissue after systemic administration of Pt(acac)₂(DMS), indicating that the compound is able to cross the BBB [10, 11] without exerting neurotoxicity [12]. This BBB permeability is likely attributed to the increased lipophilicity conferred by the ligands.

Pt(acac)₂(DMS) has shown strong cytotoxic effects in various cancer cell models, promoting cell death, mitochondrial depolarization, ATP depletion, and activation of caspases −9 and −7. Moreover, Pt(acac)₂(DMS) influences calcium-regulating proteins such as PMCA and activates key signaling pathways like PKC-α and MAPKs, which play crucial roles in cell death regulation [8, 9, 13].

More broadly, platinum-based drugs are also known to impact intracellular Ca²⁺ homeostasis essential in shaping cell responses. Calcium can trigger either survival or death pathways by activating signaling to induce autophagy or to promote apoptosis [14]. Autophagy itself, an intracellular process that recycles damaged organelles and long-lived proteins, plays a paradoxical role in cancer. On one hand, it protects cells by maintaining homeostasis and limiting genomic damage, acting as a tumor suppressor. On the other hand, it can help cancer cells survive under stress conditions, such as nutrient deprivation or chemotherapy, thereby contributing to resistance [15]. In GB, autophagy is particularly relevant for tumor adaptation and therapeutic evasion. Its interaction with apoptosis is controlled by shared regulators, including Beclin-1, the Bcl-2 protein family, and p62/SQSTM1, which together fine-tune the balance between survival and cell death [16, 17].

In this study, we compare the effects of CDDP and Pt(acac)₂(DMS) in U251 glioblastoma cells. Using a range of techniques, including biochemical assays, flow cytometry, immunocytochemistry, and ultrastructural analysis, we explore how these drugs influence cell death pathways, with particular focus on autophagy. A better understanding of these responses could reveal novel strategies to overcome GB resistance and improve therapeutic outcomes.

## RESULTS

### Dose–response analysis of cell viability and cell cycle progression

**Fig. 1:**
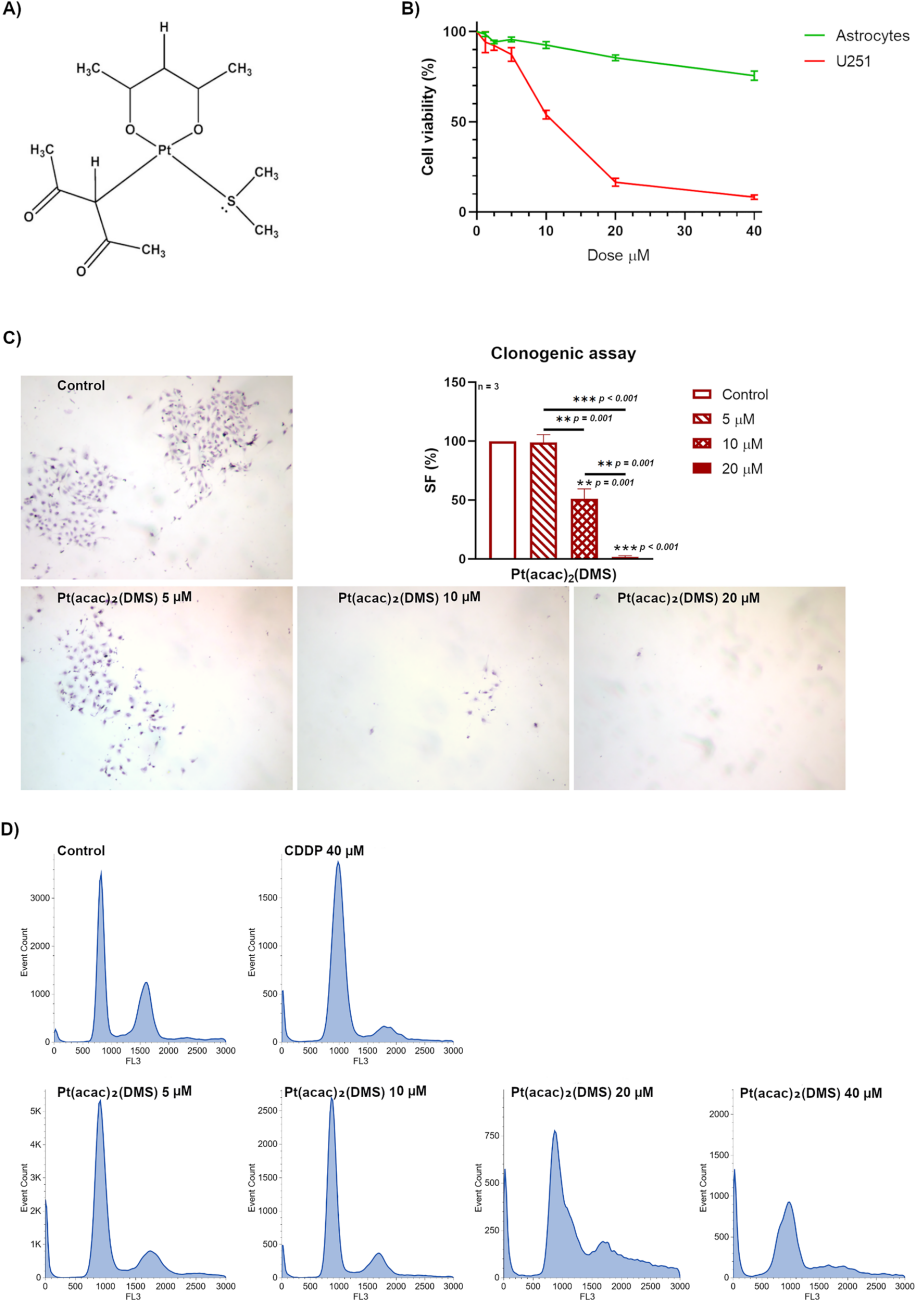
Pt(acac)₂(DMS) reduces glioblastoma cell viability, impairs clonogenic survival, and induces cell-cycle alterations in a dose-dependent manner. **A** [Pt(O,O′-acac)(γ-acac)(DMS)] (Pt(acac)₂(DMS)) molecular structure. **B** Curve representing viability of U251 and human astrocytes obtained with MTT assay after 48 h of acute exposure to increasing Pt(acac)₂(DMS) (0–40 μM) concentrations. The relative cell viability is expressed as a percentage relative to the untreated control cells. **C** Representative table of U251 cells, showing colony formation after 10 days from the 48 h of treatments with Pt(acac)₂(DMS) 5, 10, or 20 μM (magnification 4×) and the relative histogram of the mean survival fraction expressed as percentage (SF%) ± SEM, significance of differences as for the ordinary one-way ANOVA test: *p < 0.05; **p < 0.01; ***p < 0.001, where * indicates the difference of Pt(acac)₂(DMS) versus the control condition. (D) Cytofluorimetric histograms of U251 DNA content after PI staining in control cells, 48 h CDDP 40 μM treated cells, 48 h Pt(acac)₂(DMS) treated cells at different concentrations (5, 10, 20, or 40 μM).

The cytotoxic effects of the platinum(II) complex [Pt(O,O′-acac)(γ-acac)(DMS)], in this work indicated as Pt(acac)₂(DMS), were evaluated in both human astrocytes and U251 glioblastoma cells across a range of concentrations (0–40 μM) (Fig. 1B). In astrocytes, treatment with the compound induced a moderate, dose-dependent decrease in cell viability, with values decreasing from 100% in untreated controls to approximately 75.5% at the highest concentration. Notably, astrocyte viability remained relatively high at 10 μM, with 92.5% ± 1.88% of cells remaining viable. In contrast, U251 glioblastoma cells exhibited a markedly higher sensitivity to the compound. While cell viability was only modestly affected at lower concentrations (e.g., 92.2% ± 2.73% at 2.5 μM), a significant reduction was observed at 10 μM, with viability dropping to 53.96% ± 2.33%. This pronounced cytotoxic effect became even more evident at higher doses.

The ten-day clonogenic assay confirmed the cytotoxic profile observed in short-term viability assays (Fig. 1C). At the lowest tested concentration of Pt(acac)₂(DMS) (5 μM), a slight reduction in the SF% of U251 cells was noted; however, this decrease was not statistically significant. A marked and statistically significant impairment (p < 0.01) of clonogenic potential was observed starting from 10 μM, with the SF reduced to 51.19% ± 2.02%. This effect became even more pronounced at 20 μM, where the surviving fraction dropped dramatically to 1.79% ± 0.77%.

Cell cycle analysis was performed by propidium iodide staining to evaluate the effects of Cisplatin (CDDP) and Pt(acac)₂(DMS) (Fig. 1D). After 48 h of exposure to 40 µM CDDP, the SubG1 population increased to 8.4% compared to 2.7% in untreated control cells, accompanied by an increase in G1 phase (73.6%) and a reduction in S phase (5.1%), G2/M (9.3%). Treatment with Pt(O,O′-acac)(γ-acac)(DMS) at increasing concentrations (5–40 µM) resulted in a dose-dependent modification. The G1 phase initially increased from 59.5% at 5 µM to 66.8% at 10 µM, then decreased to 40.3% at 20 µM and remained stable at 44.0% at 40 µM. S phase percentages remained relatively low and stable at all concentrations, ranging from 5.6% to 6.4%, except for a marked increase to 17.1% at 20 µM. G2/M phase decreased gradually with increasing dose: from 16.4% at 5 µM to 8.7% at 40 µM.

### Time-response analysis of cell cycle progression and mitochondrial membrane potential

**Fig. 2:**
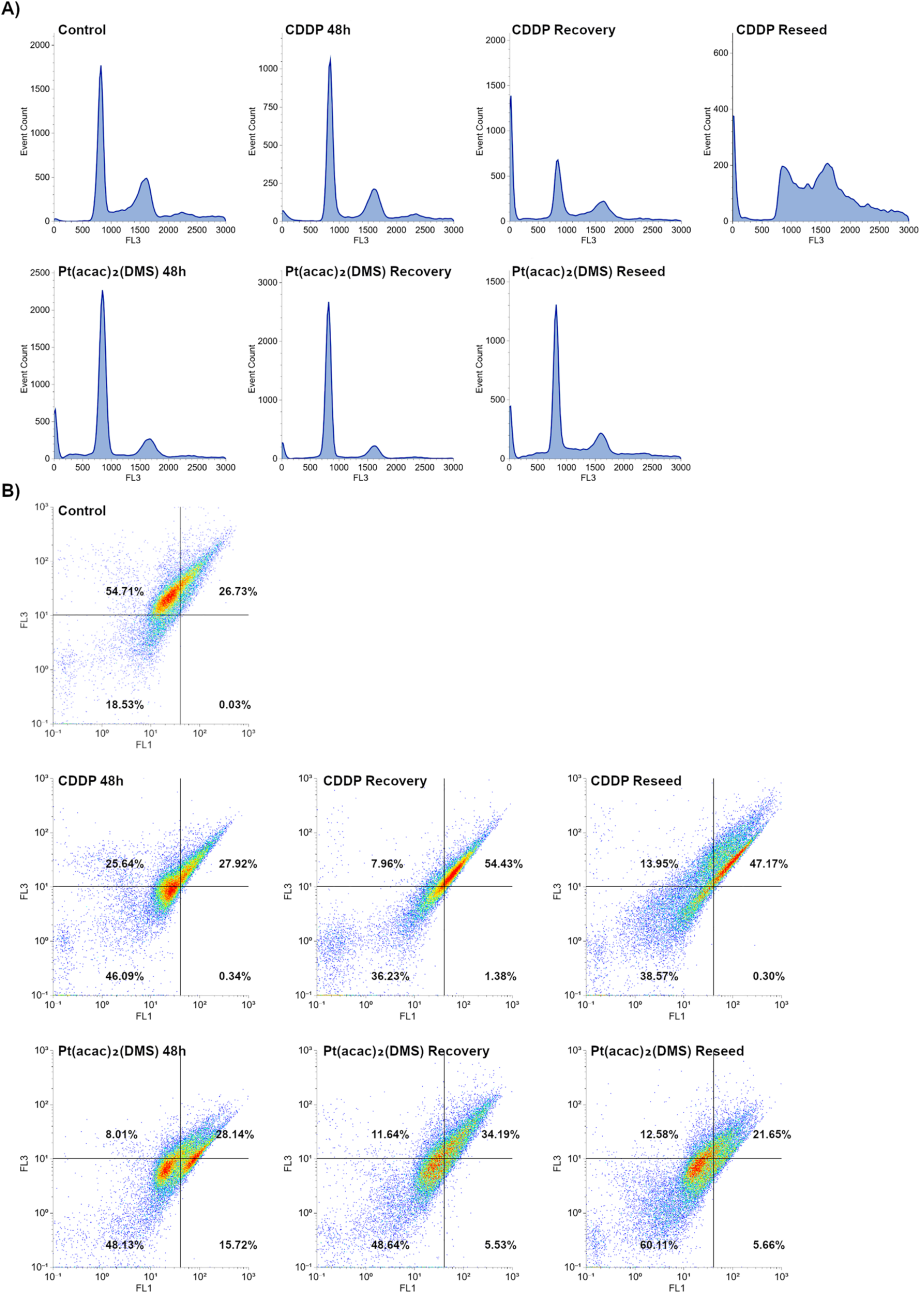
Pt(acac)₂(DMS) induces persistent cell-cycle alterations and mitochondrial dysfunction in glioblastoma cells. **A** Cytofluorimetric histograms of U251 DNA content after PI staining in control cells, 48 h CDDP 40 μM treated cells, 48 h Pt(acac)₂(DMS) 10 μM treated cells, and respective recovered and reseeded cells. **B** Scatter plots of U251 cytometric analysis of green (FL1)-red (FL3) fluorescence of JC-1 in control, 48 h CDDP 40 μM treated cells, 48 h Pt(acac)₂(DMS) 10 μM treated cells, respective recovered and reseeded cells.

In the time-dependent experiment, cells were analyzed after 48 h of acute exposure to CDDP 40 µM or Pt(acac)₂(DMS) 10 µM, and subsequently after 7 days of recovery in drug-free medium, or after trypsinization and reseeding. Exposure to 40 µM CDDP for 48 h resulted in 3.7% SubG1 and 54.3% G1, while after the recovery, the SubG1 population increased to 33.2%, with a reduction in G1 (30.0%) and an increase in the polyploid population (9.7%). In the reseeding condition, SubG1 was 13.1%, G1 dropped to 16.4%, and the polyploidy region increased to 25.7%. Treatment with 10 µM Pt(acac)₂(DMS) led to 14.0% SubG1 at 48 h and 8.3% in the polyploidy region; following recovery, SubG1 was reduced to 6.7% and the polyploid population to 5.0%, while in reseeded cells, SubG1 returned to 14.6% and the polyploidy region rose to 12.5% (Fig. 2A).

Mitochondrial membrane potential was assessed by JC-1 staining and flow cytometric analysis of FL1 (green, monomeric JC-1 in depolarized mitochondria) and FL3 (red, JC-1 aggregates in polarized mitochondria) (Fig. 2B). Untreated cells displayed 0.03% FL1⁺FL3⁻ (low ΔΨm) and 54.71% FL1⁻FL3⁺ (high ΔΨm), FL1⁻FL3⁻ population was 18.53% while FL1⁺FL3⁺ was 26.73%. Following 48-h exposure to 40 µM CDDP, FL1⁺FL3⁻ cells became 0.34%, with a decrease to 25.64% FL1⁻FL3⁺; moreover, while FL1⁺FL3⁺ remained stable at 27.92%, a significant increase was registered in the FL1⁻FL3⁻ population with 46.09% cells. A similar pattern was recorded after recovery and reseeding with an increasing number of events located in the FL1⁺FL3⁺ quadrant (7.96% FL1⁻FL3⁺, 1.38% FL1⁺FL3⁻, 36.23% FL1⁻FL3⁻, 54.43% FL1⁺FL3⁺ and 13.95% FL1⁻FL3⁺, 0.30% FL1⁺FL3⁻, 38.57% FL1⁻FL3⁻, 47.17% FL1⁺FL3⁺, respectively). Treatment with 10 µM Pt(acac)₂(DMS) induced a stronger depolarization, with FL1⁺FL3⁻ cells reaching 15.72% at 48 h, 5.53% after recovery, and 5.66% in reseeded samples. Significantly, an increment in FL1⁻FL3⁻ population was recorded, from 48.13% after 48H of continuous treatment to 60.11% in the reseeded condition; on the contrary, FL1⁻FL3⁺ events remained stable at low percentages (8.01–12.58%).

### Immunostaining analysis of Beclin-1 and Calmodulin modulation during treatment, recovery, and reseeding

**Fig. 3:**
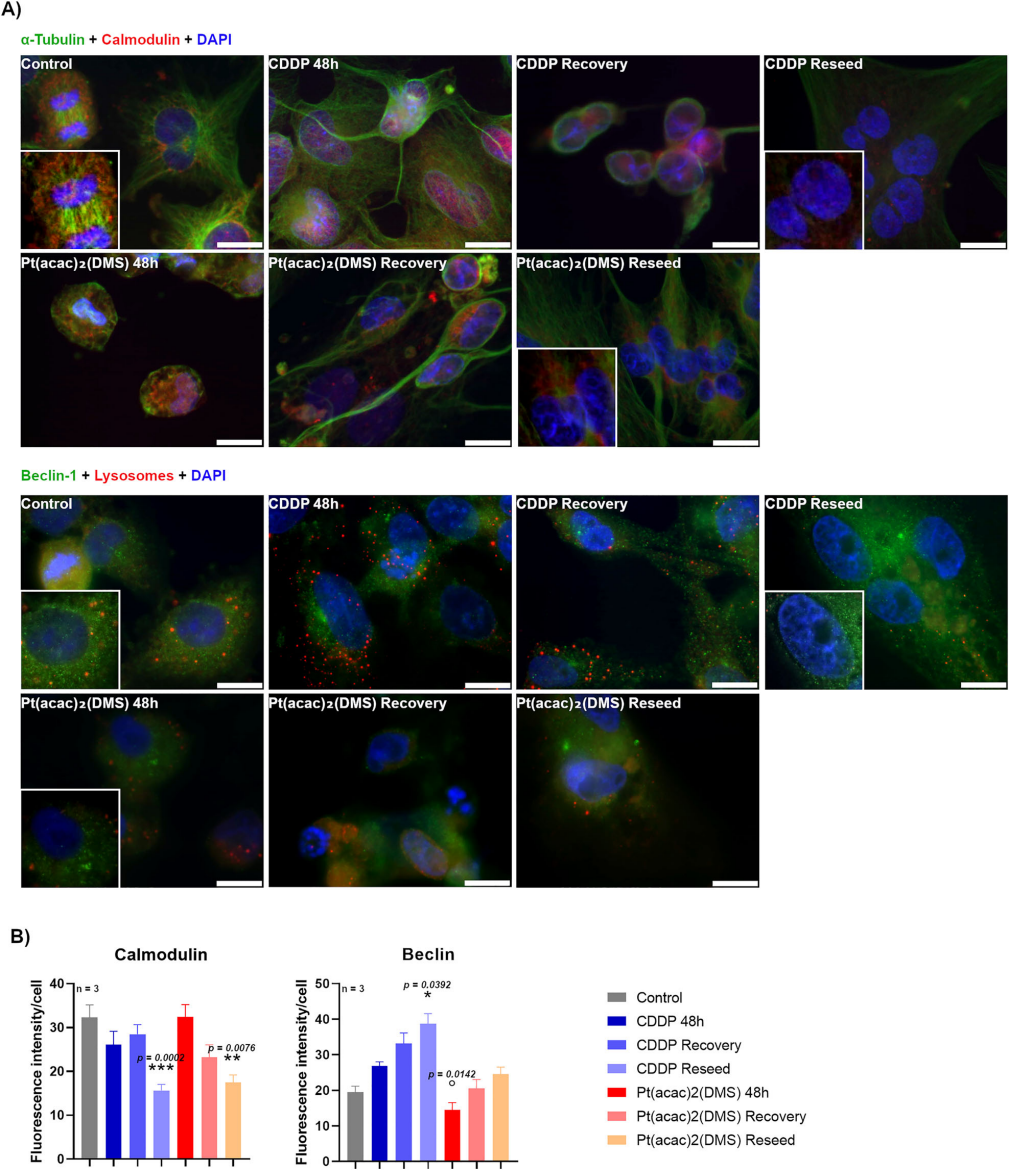
Pt(acac)₂(DMS) alters calmodulin and Beclin-1 levels during treatment, recovery, and reseeding, revealing long-term cellular stress and sustained impairment of autophagy. **A** Fluorescence microscopy. Double immunocytochemical detection of Calmodulin (red) and α-tubulin (green) or Beclin-1 (green) and lysosomes (red) in control cells, 48 h CDDP 40 μM treated cells, 48 h Pt(acac)₂(DMS) 10 μM treated cells, and respective recovered and reseeded cells. DNA was counterstained with Hoechst 33258 (blue). Bar 20 μm. **B** Histograms showing the mean fluorescence intensity value of the immunolabelling ± SEM for Calmodulin or Beclin-1, significance of differences as for the ordinary one-way ANOVA test (Calmodulin) and Kruskal–Wallis test (Beclin-1): *p < 0.05; **p < 0.01; ***p < 0.001, where * indicates the difference of Pt(acac)₂(DMS) or CDDP versus the control condition and ° indicates the difference of Pt(acac)₂(DMS) versus CDDP.

Following 48-h acute exposure to chemotherapeutic agents and a subsequent 7-day recovery phase in drug-free medium before reseeding, Beclin and Calmodulin expression levels were evaluated (Fig. 3). Beclin-1 is a key initiator of autophagosome formation, and its activity is modulated by calmodulin, a calcium-binding messenger protein that can influence autophagic signaling pathways.

Calmodulin showed a diffuse cytoplasmic and perinuclear fluorescence, reflecting its broad role in calcium signaling throughout the cell in the controls, in the recovered and reseeded cells from both CDDP and Pt(acac)₂(DMS) groups, an alerted signal distribution was observed. Calmodulin signal was concentrated in the perinuclear area. The levels of this marker in control cells were 32.27 (±2.89). After 48 h, expression dropped to 26.09 (±3.07) in CDDP-treated and remained stable at 32.41 (±2.83) in Pt(acac)₂(DMS)-treated cells. After 7 days of recovery, values slightly increased in CDDP (28.47 ± 2.17) and decreased in Pt(acac)₂(DMS) (23.23 ± 2.82). However, a marked reduction emerged at the reseed stage, with values of 15.64 (±1.37) in CDDP and 17.47 (±1.73) in Pt(acac)₂(DMS)-treated cells.

While neither acute treatment nor recovery induced significant changes in Calmodulin versus control, significant downregulation emerged in both “Reseed” conditions. Calmodulin levels were significantly lower in the CDDP Reseed group (−51.5% vs. control, p = 0.0002) and Pt(acac)₂(DMS) Reseed group (−45.9% vs. control, p = 0.0076). The drop in Calmodulin from the recovery to the reseed stage was significant for CDDP (p = 0.0023) and for Pt(acac)₂(DMS) when comparing reseed vs. 48 h (p = 0.0175), while no statistical significance was reached comparing CDDP 48 h and the reseed condition (−51.5%, p = 0.1523).

Beclin staining revealed a diffuse cytoplasmic fluorescence in the control condition; occasional punctate signals, probably corresponding to pre-autophagosomal structures, appeared in Pt(acac)₂(DMS) treated groups, while a more extensive dot-like pattern emerged in samples exposed to CDDP. Beclin expression in untreated control cells was 19.50 (±1.66). Treatment with CDDP resulted in a progressive increase in the measured immunopositivity from 26.84 (±1.16) after 48 h to 38.71 (±2.83) (+37.6–70%) following reseeding, with the latter showing a statistically significant increase compared to control (p = 0.0392). In contrast, Pt(acac)₂(DMS) induced a lower response at 48 h (14.47 ± 2.03), which remained at basal levels even after recovery and reseeding (20.48 ± 2.54 and 24.59 ± 1.92, respectively). The latter condition showed a modest increase (+26.1% vs control), though it wasn’t statistically significant. Direct comparison between CDDP and Pt(acac)₂(DMS) at 48 h revealed a significant difference (p = 0.0142).

### Immunostaining analysis of autophagic flux and compartmentalized LC3b/p62 expression during treatment, recovery, and reseeding

**Fig. 4:**
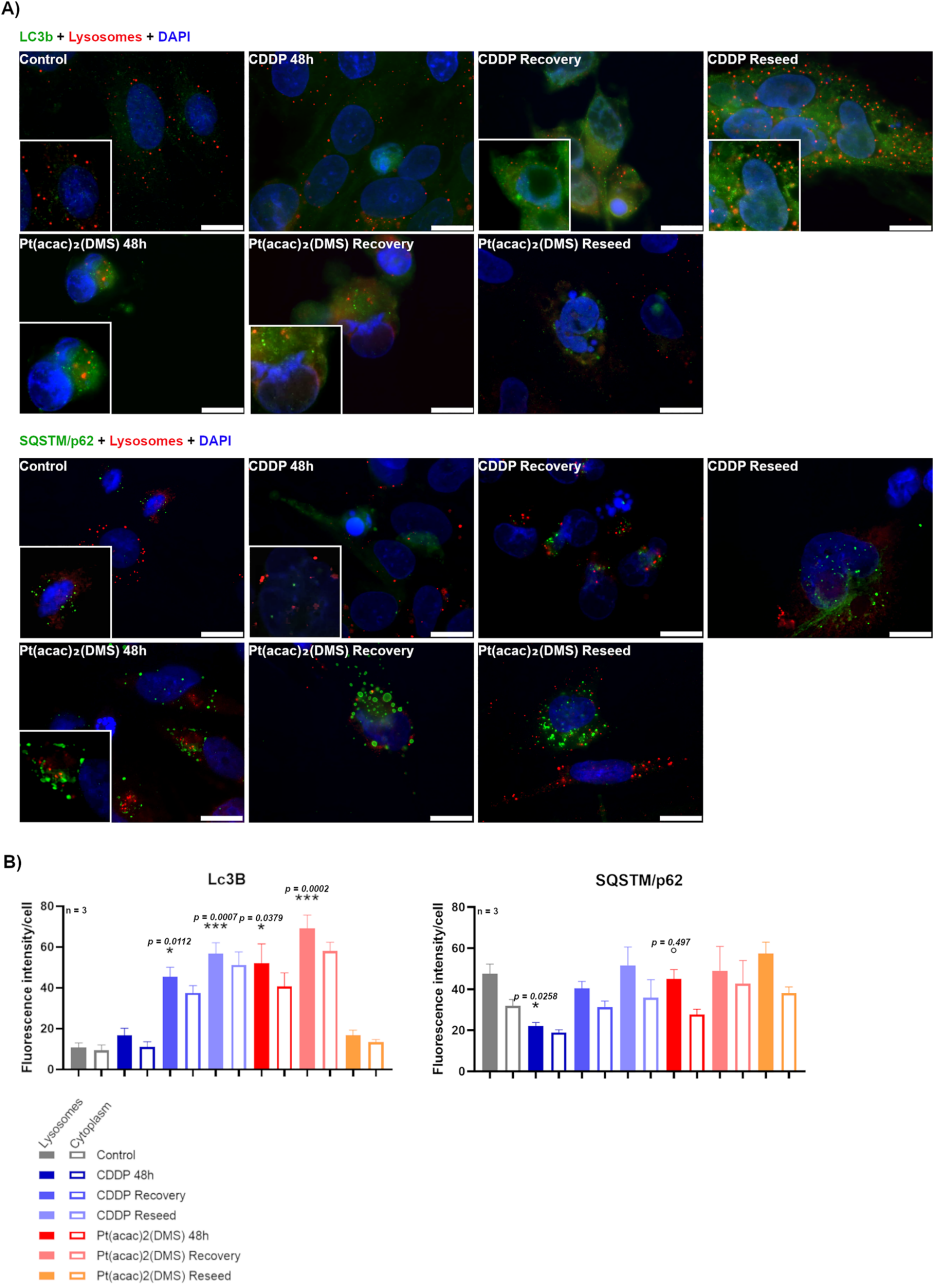
Pt(acac)₂(DMS) modulates LC3b and p62 during treatment, recovery, and reseeding, revealing an incomplete autophagic response. **A** Fluorescence microscopy. Double immunocytochemical detection of LC3b or SQSTM/p62 (green) and lysosomes (red) in control cells, 48 h CDDP 40 μM treated cells, 48 h Pt(acac)₂(DMS) 10 μM treated cells, and respective recovered and reseeded cells. DNA was counterstained with Hoechst 33258 (blue). Bar 20 μm. **B** Histograms showing the mean fluorescence intensity value of the immunolabelling ± SEM for LC3b or SQSTM/p62, significance of differences as for the ordinary one-way ANOVA test (LC3b) and Kruskal–Wallis test (SQSTM/p62): *p < 0.05; **p < 0.01; ***p < 0.001, where * indicates the difference of Pt(acac)₂(DMS) or CDDP versus the control condition and ° indicates the difference of Pt(acac)₂(DMS) versus CDDP.

Following CDDP or Pt(acac)₂(DMS), LC3b and p62/SQSTM1 expression were quantified in the lysosomes and in the cytoplasmic compartment (Fig. 4). LC3B is a marker of autophagosomes; during autophagy, LC3B-I is converted to LC3B-II, which is associated with the autophagosomal membrane. p62/SQSTM1 serves as an autophagy receptor linking ubiquitinated cargo to LC3B for degradation and is itself degraded in autolysosomes.

In the lysosomal fraction, LC3b in control cells was 10.80 (±2.22). After CDDP recovery and reseed phases, lysosomal LC3b rose to 45.40 (±4.72) and 56.79 (±5.30), respectively. Pt(acac)₂(DMS) induced a peak already at 48 h (52.33 ± 9.22) and even higher at recovery (69.30 ± 6.38), returning to near baseline at reseed (16.70 ± 2.60).

In the cytoplasmic compartment, LC3b increased after CDDP recovery (37.66 ± 3.44) and reseeding (51.24 ± 6.35), while Pt(acac)₂(DMS) led to 58.18 (±4.18) at recovery and returned to 13.41 (±2.55) at reseeding. Control values were 9.34 ( ± 2.68).

Lysosomal LC3b significantly increased after CDDP recovery (p = 0.0112) and reseeding (p = 0.0007) compared to control. Similarly, Pt(acac)₂(DMS) induced a marked lysosomal LC3b increase after 48 h (p = 0.0379) and recovery (p = 0.0002). LC3b levels after Pt(acac)₂(DMS) reseeding were not significantly different from control, suggesting a transient induction. Cytoplasmic LC3b also increased significantly post-CDDP recovery (p = 0.043) and reseed (p = 0.002), and after Pt(acac)₂(DMS) recovery (p = 0.0004). Notably, the difference between CDDP 48 h and reseed phases was significant in both lysosomal (p = 0.0091) and cytoplasmic compartments (p = 0.0069). For Pt(acac)₂(DMS), a significant reduction in LC3b was observed between recovery and reseed stages in both lysosomal (p = 0.0199) and cytoplasmic compartments (p = 0.0397).

In the samples, p62/SQSTM1 showed moderate cytoplasmic fluorescence, with evident dots when autophagy is activated. In the conditions following Pt(acac)₂(DMS) washout, p62-positive puncta became larger and more prominent, suggesting the accumulation of undegraded autophagic cargo. In lysosomal fractions, p62/SQSTM1 levels were significantly decreased following 48 h exposure to CDDP compared to untreated control (22.19 ± 1.64 vs. 47.55 ± 4.74; p = 0.0258). In contrast, treatment with Pt(acac)₂(DMS) for 48 h did not significantly affect lysosomal p62/SQSTM1 levels compared to control (45.09 ± 4.55; p > 0.9999). Direct comparison between the two compounds revealed a significant difference in lysosomal p62/SQSTM1 levels with p = 0.0497. Following drug washout and reseeding, p62/SQSTM1 levels increased to 51.72 (±8.93) and 57.42 (±5.52), respectively, for reseeded cells after CDDP and Pt(acac)₂(DMS). Results have been validated through an autophagic flux assay using a well-established inhibitor [18] (Fig. S1).

**Fig. 5:**
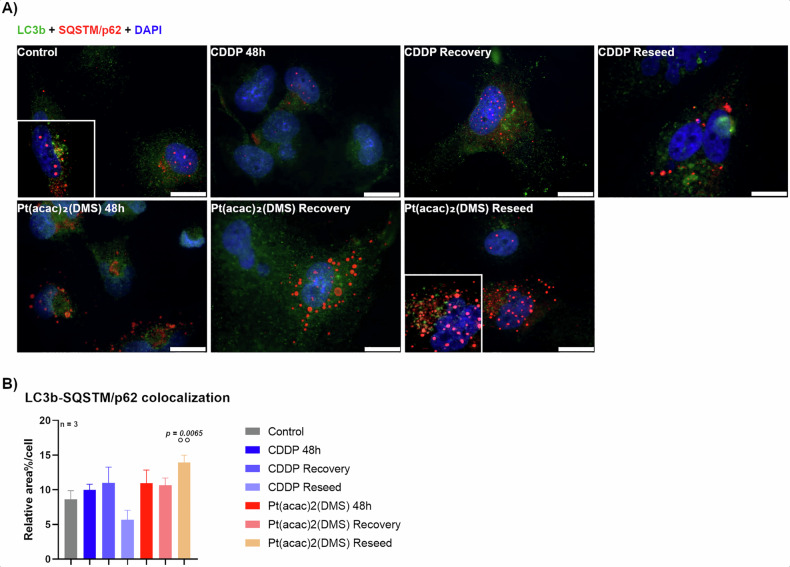
Pt(acac)₂(DMS) increases LC3b–p62 colocalization, indicating inefficient lysosomal degradation during treatment and recovery. **A** Fluorescence microscopy. Double immunocytochemical detection of LC3b (green) and SQSTM/p62 (red) in control cells, 48 h CDDP 40 μM treated cells, 48 h Pt(acac)₂(DMS) 10 μM treated cells, and respective recovered and reseeded cells. DNA was counterstained with Hoechst 33258 (blue). Bar 20 μm. **B** Histograms showing the mean area of colocalization (expressed as percentage of the whole cellular area) ± SEM of LC3b and SQSTM/p62, significance of differences as for the ordinary one-way ANOVA test: **p < 0.01; where * indicates the difference of Pt(acac)₂(DMS) or CDDP versus the control condition and ° indicates the difference of Pt(acac)₂(DMS) versus CDDP.

To investigate autophagic flux in U251 glioblastoma cells, we quantified the percentage of p62/LC3b colocalization relative to the total signal across different conditions (Fig. 5). In untreated control cells, the colocalization signal was 8.65 (±1.21). Following 48 h of CDDP treatment, this slightly increased to 10.00 (±0.80) and remained stable at 10.99 (±2.28) after recovery. A decrease was observed after reseeding (5.67 ± 1.38), though not statistically significant.

Cells treated with Pt(acac)₂(DMS) had colocalization values of 10.95 (±1.90) at 48 h, 10.67 (±1.02) at recovery, and 13.94 (±1.05) after reseeding. Although no significant differences were found across timepoints within this treatment group, reseeded cells showed significantly higher colocalization than CDDP-treated reseeded cells (13.94% vs. 5.67%, p = 0.0065), indicating a sustained autophagic response following chronic Pt(acac)₂(DMS) exposure.

### Ultrastructural evaluation by Transmission Electron Microscopy

In control cells, the typical morphology of U251 cells was observed, characterized by a nucleus with decondensed chromatin and a prominent nucleolus or multiple nucleoli, which are typical of neoplastic cells. The cytoplasm contained abundant free and membrane-bound ribosomes, granular endoplasmic reticulum, and few healthy mitochondria; lysosomes were scarce.

After 48 h of cisplatin treatment, cells displayed nuclear heteromorphism and extensive cytoplasmic vacuolization, along with an increased number of small mitochondria with depleted matrix. Upon recovery, cells exhibited typical autophagic features, including cytoplasmic segmentation, condensed chromatin, numerous lysosomes, and cytoplasmic vacuoles (autophagic bodies) containing degraded cytosolic components, particularly mitochondria. During the reseeding phase, cells nearly regained normal nuclear morphology; however, many small and swollen mitochondria persisted in the cytoplasm.

**Fig. 6:**
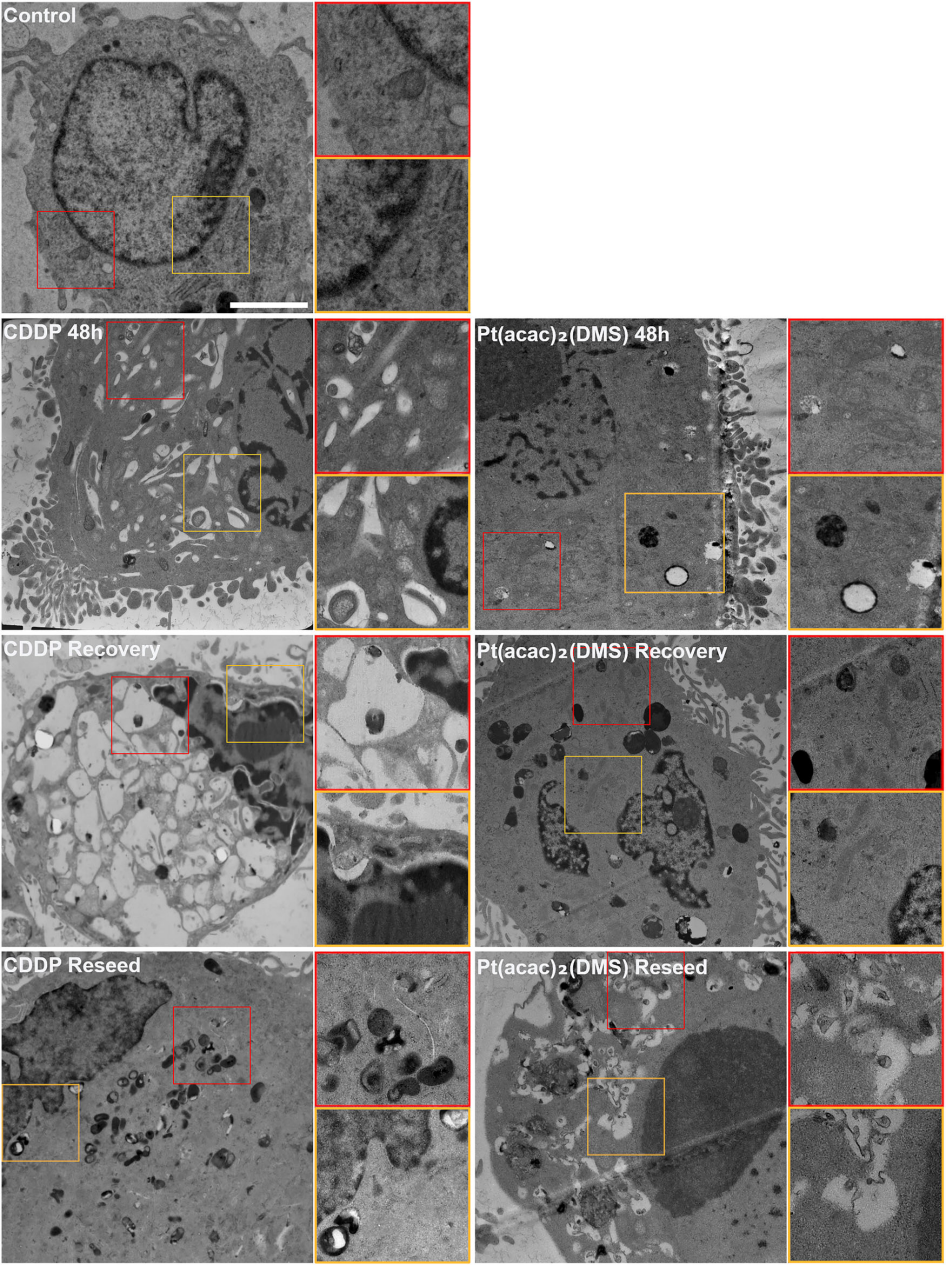
Pt(acac)₂(DMS) induces distinct ultrastructural changes. Ultrastructural morphology of 48 h CDDP 40-μM-treated cells, 48 h Pt(acac)₂(DMS) 10-μM-treated cells, and respective recovered and reseeded cells. Bar 2 μm.

In contrast, cells treated with Pt(acac)₂(DMS) for 48 h showed distinct ultrastructural changes. Mitochondria appeared elongated with a homogeneous matrix and tubular shape, a pattern that was also evident during recovery, where accumulation of damaged organelles was noted. Under reseeding conditions, cells exhibited a highly condensed nucleus along with combined autophagic and apoptotic features. The cytoplasm was densely populated with double-membrane autophagic bodies containing still clearly recognizable electron-lucent mitochondria (Fig. 6).

## Discussion

In this study, we investigated the cellular effects of Cisplatin (CDDP) and the platinum(II) complex [Pt(O,O′-acac)(γ-acac)(DMS)], indicated as Pt(acac)₂(DMS), on U251 glioblastoma cells, focusing on cell cycle dynamics, mitochondrial function, and autophagy-related markers.

Previous studies have highlighted the enhanced membrane permeability of Pt(acac)₂(DMS) compared to cisplatin [19]. Importantly, in vivo experiments have demonstrated that Pt(acac)₂(DMS) is able to cross the blood–brain barrier, overcoming a major limitation of cisplatin in brain tumor therapy [11]. However, dedicated pharmacokinetic investigations in glioblastoma in vivo models are still required to fully characterize Pt(acac)₂(DMS) distribution and therapeutic potential in brain tumors.

Moreover, unlike cisplatin, which is associated with significant systemic toxicity, Pt(acac)₂(DMS) has shown reduced neurotoxicity in preclinical rat models [20]. These features make it a compelling candidate for further investigation in glioblastoma treatment.

Our findings demonstrate that Pt(acac)₂(DMS) exerts a selective cytotoxic effect on glioblastoma cells, with significantly less impact on non-transformed astrocytes. The differential sensitivity observed between U251 glioblastoma cells and primary human astrocytes suggests a promising therapeutic window for this compound. Notably, at a concentration of 10 μM, Pt(acac)₂(DMS) induced a pronounced reduction in U251 cell viability, while maintaining over 90% viability in astrocytes. The results of the clonogenic assay further support the potency of Pt(acac)₂(DMS) in impairing the long-term proliferative capacity of glioblastoma cells, possibly preserving normal healthy cells.

Cell cycle analysis provided additional mechanistic insights into the effects of Pt(acac)₂(DMS). Treatment with increasing concentrations of the compound induced a dose-dependent alteration in cell cycle distribution. Testing a broader concentration range for Pt(acac)₂(DMS) allowed us to delineate a dose–response relationship, providing insights into sub-toxic *versus* higher-dose effects, which could be particularly valuable from a translational perspective, as it helps define the potential therapeutic window and informs future in vivo dosing strategies. At lower doses (5–10 μM), there was an accumulation of cells in the G1 phase, suggesting a G1 arrest. However, at higher concentrations (≥20 μM), an extreme shift of the histogram to the left and an S phase that is less and less distinguishable from the G1 phase were observed, indicating the induction of DNA fragmentation and possible replication stress. These effects were distinct from those observed with cisplatin, which primarily increased the G1 population and subG1 fraction, consistent with DNA damage and apoptosis induction [4]. Notably, the reduction in G2/M phase observed with Pt(acac)₂(DMS) across increasing doses further supports a disruption of cell cycle progression, potentially linked to irreparable cellular damage or activation of cell death pathways.

Time-course experiments assessing recovery and reseeding after acute exposure revealed further differences. Following CDDP treatment recovery and reseed, a decrease in SubG1 population and, on the contrary, an increase in the polyploid population indicate genomic instability [21]. The rise in polyploidy in reseeded cells from Cisplatin treatment may reflect mitotic aberrations or endoreduplication. This is notable because polyploid giant cancer cells have been implicated as a reservoir of tumor plasticity that can survive genotoxic stress, give rise to diverse progeny, and contribute to relapse and chemoresistance [22]. Conversely, Pt(acac)₂(DMS)-treated cells exhibited consistent SubG1 levels post-recovery and reseeding, suggesting different cell death kinetics and a reduced tendency to generate polyploid survivor populations.

Mitochondrial membrane potential (ΔΨm) analysis demonstrated a more pronounced mitochondrial depolarization after Pt(acac)₂(DMS) treatment compared to CDDP, with elevated FL1⁺FL3⁻ populations at all time points, including recovery and reseeding, and elevated FL1⁻FL3⁻ populations sign of a strong mitochondrial impairment. This persistent mitochondrial dysfunction may be linked to intrinsic apoptotic pathways or metabolic stress more effectively activated by Pt(acac)₂(DMS) than Cisplatin [23].

Alterations of mitochondrial functionality due to Pt(acac)₂(DMS) have already been reported in other cancerous cellular models, such as breast cancer or neuroblastoma [24, 25], where, as for glioblastoma, mitochondrial dysfunction is increasingly recognized as a key axis of therapy response and resistance.

Our fluorescence-based analysis of autophagy-related markers reveals divergent dynamics in the modulation of autophagic flux and calcium signaling components. These differences likely reflect compound-specific effects on cellular stress response and recovery capacity.

Beclin-1, a key initiator of autophagy, showed a biphasic response to CDDP. Its signal was reduced at 48 h post-treatment, suggesting an early suppression of autophagy initiation, which aligns with known acute stress-induced inhibition of Beclin-1 activity. However, Beclin-1 levels gradually recovered and reached control values in reseeded cells, indicating restoration of autophagy signaling and a potential adaptive response to drug-induced stress [26]. In contrast, Pt(acac)₂(DMS)-treated cells displayed a persistently lower Beclin-1 signal throughout all time points. This suggests a sustained impairment of autophagy, possibly reflecting a more profound or irreversible disruption of upstream signaling pathways compared to Cisplatin.

Calmodulin, a calcium-binding messenger protein involved in multiple cellular processes including autophagy and cell survival [27], remained unchanged at 48 h in both treatments but significantly decreased at later time points. The delayed downregulation may be indicative of long-term cellular stress or mitochondrial dysfunction, known to affect intracellular calcium homeostasis. The similar trend in both compounds suggests that calcium signaling dysregulation is a shared outcome.

LC3B, a marker of autophagosome formation [28], exhibited compound-specific trends. CDDP induced a gradual increase in LC3B levels over time, despite no initial change at 48 h. This suggests a delayed yet sustained autophagic response, potentially linked to cellular efforts to clear damaged components and restore homeostasis to allow survival in a subset of cells. Conversely, Pt(acac)₂(DMS) led to an early increase in LC3B that declined to baseline after reseeding. This transient activation, followed by normalization, could indicate either a short-lived autophagic response or a block in later stages of autophagy leading to feedback suppression of LC3B expression [29].

p62/SQSTM1, a selective autophagy substrate, was reduced after 48 h of Cisplatin exposure, consistent with autophagic degradation. Its subsequent increase toward basal levels in reseeded cells suggests autophagy resolution or flux inhibition during recovery [29]. In Pt(acac)₂(DMS)-treated cells, p62 levels remained stable and slightly increased, further supporting the hypothesis of an incomplete or inefficient autophagic response.

Interestingly, the colocalization between p62 and LC3B, a surrogate for autophagosome cargo recognition, remained generally stable, implying that the formation of autophagic complexes is not grossly disrupted by either compound. However, a divergent trend was noted in the reseeded condition: a decrease in colocalization in CDDP-treated cells and an increase in Pt(acac)₂(DMS)-treated cells. These opposite trends may reflect distinct regulatory mechanisms in autophagosome maturation and cargo turnover. Specifically, the decrease in Cisplatin-treated reseeded cells could be linked to enhanced autophagic clearance, whereas the increase in Pt(acac)₂(DMS) samples may suggest accumulation due to inefficient degradation [30].

Taken together, our findings demonstrate that both Cisplatin and Pt(acac)₂(DMS) induce stress responses in U251 glioblastoma cells, with distinct biological consequences. Cisplatin tends to produce polyploid survivor populations that are linked to tumor plasticity and later relapse, whereas Pt(acac)₂(DMS) produces profound mitochondrial damage and signs of defective autophagic clearance that may reduce the capacity of surviving cells to repopulate. Specifically, Pt(acac)₂(DMS) appears to trigger an incomplete or inefficient autophagic response. This is also supported by the accumulation of damaged organelles and partially degraded mitochondria observed ultrastructurally, particularly in reseeded Pt(acac)₂(DMS)-treated cells, corroborating the notion of inefficient autophagic clearance. In contrast, Cisplatin-treated cells exhibit more dynamic autophagic responses with evidence of flux progression and cytoplasmic remodeling during recovery. While further evaluation of autophagic flux with specific inhibitors is warranted to better differentiate if the observed effects are related to a straightforward autophagy block or a two-step process involving activation followed by inhibition, these data suggest that Pt(acac)₂(DMS) may limit cells’ capacity to adapt to chemotherapeutic stress, potentially diminishing their ability to develop resistance mechanisms. This impaired adaptive response may contribute to the compound’s distinct cytotoxic profile and supports its potential utility in overcoming resistance pathways typically engaged during standard chemotherapy, thereby offering renewed hope for the development of more effective glioblastoma therapies. Nonetheless, further studies are necessary to validate these findings in additional glioblastoma cell lines and to elucidate the molecular interplay between autophagy, organelle homeostasis, and stress signaling pathways through targeted molecular biology approaches. Furthermore, to assess the translational relevance of these results, in vivo studies will be essential. Given that chemotherapy alone is no longer the standard of care in glioblastoma, evaluating this compound (or others that modulate autophagic clearance) in combination with radiotherapy or immunotherapy, approaches currently of high clinical interest, will be crucial. Investigating the effects of such combinations on the tumor microenvironment, immune response, overall survival, and quality of life will also be vital to fully understand the broader implications of autophagy modulation in glioblastoma therapy.

## Materials and methods

### Cell culture and treatments

Human glioblastoma U251 cells (Sigma-Aldrich, Milan, Italy) were cultured in 75 cm² flasks in minimal essential medium (MEM) supplemented with 10% fetal bovine serum, 1% glutamine, and 100 U/mL penicillin-streptomycin, maintained at 37 °C in a humidified 5% CO₂ atmosphere. Primary cultures of normal human astrocytes, kindly provided by Prof Paolillo Mayra (Department of Drug Sciences, University of Pavia), served as healthy controls. Astrocytes were maintained in DMEM-F12 (Dulbecco’s Modified Eagle Medium and Ham’s F-12 Nutrient Mixture) supplemented with 10% FBS, 1% L-glutamine, and 1% penicillin-streptomycin, under identical incubation conditions (37 °C, 5% CO₂). All reagents were sourced from Celbio S.p.A. and EuroClone S.p.A. (Pero, Milan, Italy). Cells have been tested at regular intervals for mycoplasma contamination (MycoStrip®, InvivoGen, Aurogene SRL, Rome, Italy). Twenty-four hours prior to experiments, cells were seeded on glass coverslips for fluorescence microscopy or cultured in 75 cm² flasks for flow cytometry analysis. Cells were treated as described above; all the reagents used have been solubilized in the culture medium.

### MTT viability assay

Cell viability was assessed using MTT salt (CAS 298-93-1, Calbiochem, Inalco, Italy). Single cells were seeded at a density of 5 × 10^4^ cells per well in 96-well plates. After 24 h, cells were treated with the respective compounds. Control cells were incubated with fresh culture medium and vehicle only. Concentration ranges of 0–40 μM for Pt(acac)₂(DMS) were tested. Following 48 h of treatment, the culture medium was replaced with fresh medium containing a 1:10 dilution of 5 mg/mL MTT solution in sterile 1× PBS (EuroClone). After a 3-h incubation at 37 °C, the formazan crystals formed were dissolved in 100 μL of DMSO per well. Absorbance was measured at 490 nm using the Elx808™ Absorbance Microplate Reader (BioTek Instruments Inc., Winooski, Vermont, USA).

### Clonal cell survival assay

U251 cells were treated with 5 μM, 10 μM, or 20 μM of Pt(acac)₂(DMS) for 48 h. Subsequently, cells were detached using 0.10% trypsin–EDTA, plated at low density in six-well plates, and incubated for 10 days in drug-free medium to allow colony formation. After this 10-day time window, the colonies were gently washed with PBS, fixed, and stained with Hematoxylin. Subsequently stained colonies, defined as groups of ≥50 cells [31, 32], were counted using an Olympus CKX41 inverted microscope equipped with an Olympus MagnaFire digital camera.

Plating efficiency-based (PE) evaluation of survival fractions was calculated as previously reported [33]. Briefly, PEs were calculated by dividing the number of colonies obtained by the number of cells seeded under untreated conditions. Subsequently, surviving fractions (SF) were determined by dividing the number of colonies obtained by the number of cells seeded at a given Pt(acac)₂(DMS) concentration multiplied by PE. Plating efficiency (PE) and survival fraction (SF) were calculated using the following formulas:

PE% = n. of colonies formed/n. of cells seeded × 100%

SF = n. of colonies formed after Pt(acac)₂(DMS) exposure/(n. of cells seeded × PE).

### Flow cytometry

U251 cells were treated with 40 μM of cisplatin, or 5 μM, 10 μM, 20 μM, or 40 μM of Pt(acac)₂(DMS) for 48 h during one experimental setting, and according to the following protocols for another experimental setting: (i) incubation with 40 μM CDDP or 10 μM Pt(acac)₂(DMS) for 48 h, (ii) incubation with 40 μM CDDP or 10 μM Pt(acac)₂(DMS) for 48 h, followed by a 7-day recovery period in drug-free medium, (iii) Incubation with 40 μM CDDP for 48 h, followed by 7 days recovery in drug-free medium, then reseeding and growth in normal medium for an additional 4 days. The CDDP concentration was chosen based on previous publications [34, 35], in particular, as reported, the same concentration used here reduced cell viability by approximately 50% (IC₅₀) in the same cell line [32]. Pt(acac)₂(DMS) concentration was selected based on the reported MTT assay. After treatments, cells were detached by mild trypsinization to obtain single-cell suspensions, washed with phosphate-buffered saline (PBS), and permeabilized in 70% ethanol for 10 min at room temperature. After permeabilization, cells were stained with Propidium Iodide (PI; 50 μg/mL, Sigma-Aldrich), in a solution containing RNase A 100 U/mL, for 10 min at room temperature and analyzed by flow cytometry (Partec PAS III, Münster, Germany) using an argon laser (488 nm excitation) and 610 nm long-pass filter for PI fluorescence detection.

Mitochondrial membrane potential was assessed using the JC-1 dye (5,5′,6,6′-tetrachloro-1,1′,3,3′-tetraethylbenzimidazolcarbocyanine iodide; Molecular Probes, Invitrogen, Thermo Fisher Scientific, Monza, Milan, Italy). JC-1 exhibits potential-dependent fluorescence emission shifts from red (polarized mitochondria) to green (depolarized mitochondria). Cells were harvested by mild trypsinization, incubated with 2 μM JC-1 for 20 min at 37 °C in the dark, washed twice with PBS, and analyzed by flow cytometry (Partec PAS III) using 488 nm excitation and emission filters at 530/30 nm (green) and 585/42 nm (red). Data analysis was performed using Flowmax (Partec, version 2.70).

### Immunofluorescence staining

Cells grown on coverslips were treated according to the following protocols: (i) incubation with 40 μM CDDP or 10 μM Pt(acac)₂(DMS) for 48 h, (ii) incubation with 40 μM CDDP or 10 μM Pt(acac)₂(DMS) for 48 h, followed by a 7-day recovery period in drug-free medium, (iii) Incubation with 40 μM CDDP for 48 h, followed by 7 days recovery in drug-free medium, then reseeding and growth in normal medium for an additional 4 days. This design was chosen to assess the restoration of biological function over longer recovery periods, which is necessary to distinguish transient from persistent drug sensitivity, which may possibly reflect in vivo relapse or persistence [7, 36].

After treatments, cells were fixed with 4% formaldehyde for 20 min at room temperature, then with 70% ethanol at −20 °C for at least 24 h. Primary antibodies (Table 1) were incubated for 1 h at room temperature in a humidified chamber. After three PBS washes, secondary antibodies were applied for 60 min at room temperature, diluted 1:200 (Alexa 594 or 488 conjugated anti-mouse or anti-rabbit, or anti-human antibodies from Alexa Fluor, Molecular Probes, Invitrogen). Nuclei were counterstained with Hoechst 33258 0.1 μg/mL (Sigma-Aldrich, Milano, Italy) for 6 min, washed, and mounted with Mowiol (Calbiochemus). In order to better investigate possible alterations in the autophagic flux, an autophagic flux assay was performed using a specific inhibitor (Reagent A, Muse® Autophagy LC3-antibody Based Kit, MCH200109, Luminex Corporation, Austin, TX, USA), as published in Slivinschi et al. [18]. Fluorescence was observed using an Olympus BX51 microscope equipped with filters optimized for Hoechst, Alexa 488, and Alexa 594 fluorescence. Images were recorded with an Olympus MagnaFire camera system and processed with the Olympus Cell F software (version 3.1). Time exposure during acquisition was maintained constant for all the experimental groups to make fluorescence intensity comparable between different experimental conditions. The fluorescence intensity was subsequently analyzed using ImageJ software 1.51 (NIH, Bethesda, MD, USA) as previously published [6].

**Table 1:** Primary antibodies used for immunocytochemical studies.

Antigen	Antibody details	Dilution
Calmodulin	Abcam, rabbit monoclonal Cat. # ab199558	1:50
Beclin-1	Cell Signaling Technology, rabbit polyclonal Cat. # 3738	1:100
LC3B	Cell Signaling Technology, rabbit polyclonal Cat. # 2775	1:400
p62/SQSTM1	Abcam, mouse monoclonal Cat. # ab56416	1:100
Human autoimmune serum for lysosomes detection (*)	Kind gift of IRCCS Policlinico San Matteo, Pavia (Italy)	1:500
α-Tubulin	Invitrogen, mouse monoclonal Cat. # 62204	1:100

(*) Bottone et al. [37].

### Transmission electron microscopy (TEM)

For ultrastructural analysis, samples were fixed in 2.5% glutaraldehyde (Polysciences, Inc., Warrington, PA, USA) in culture medium for 2 h, followed by post-fixation with 1% osmium tetroxide (Sigma Chemical Co., St. Louis, MO, USA) for 2 h at room temperature. Samples were then dehydrated through a graded acetone series (30–100%) and embedded in epoxy resin EM-bed812 (Electron Microscopy Sciences, Hatfield, PA, USA). Ultrathin sections (~70 nm) were cut using a Reichert OM-U3 ultramicrotome and collected on nickel grids and counterstained with uranyl acetate for 10 min and with lead citrate for 3 min. Sections were observed under a JEM 1200 EX II (JEOL, Peabody, MA, USA) electron microscope operating at 100 kV and equipped with a MegaView G2 CCD camera (Olympus OSIS, Tokyo, Japan) [6].

### Statistical analysis

Each experiment was carried out as three independent replicates, as for the G-power analysis. Quantitative data were analyzed using GraphPad Prism version 8.0 (GraphPad Software Inc., San Diego, CA, USA). Results are presented as mean ± standard error of the mean (SEM). The normality of parameter distributions was assessed using the D’Agostino & Pearson, Anderson–Darling, Kolmogorov–Smirnov, and Shapiro–Wilk tests. Subsequently, data were analyzed to identify statistically significant differences. For data that did not meet the normality criteria, the Kruskal–Wallis test was applied. Conversely, for normally distributed data, one-way ANOVA was performed, followed by Tukey’s post hoc test. A *p*-value of less than 0.05 was considered statistically significant.

## Supplementary information

Supplementary Figure S1 caption

Figure S1

## Data Availability

The original contributions presented in this study are included in the article. Further inquiries can be directed to the corresponding author.
