# Supplementary material for: In vitro cytotoxic mechanisms of Pt(O,O′-acac)(γ-acac)(DMS): mitochondrial dysfunction and impaired autophagy in U251 cell line
Source: Cell Death Discov. 2026 Jan 9;12:79. doi: 10.1038/s41420-025-02918-7 (PMC12877171; doi:10.1038/s41420-025-02918-7)
Supplement: Supplementary file 1 — Supplementary Figure S1 caption [file 41420_2025_2918_MOESM1_ESM.docx]

**Figure S1.** Fluorescence microscopy. Double immunocytochemical detection of LC3b (green) and SQSTM/p62 (red) in control and autophagic flux inhibitor-treated cells. DNA was counterstained with Hoechst 33258 (blue). Bar 50 μm.
