# Supplementary figures and images for: In vitro cytotoxic mechanisms of Pt(O,O′-acac)(γ-acac)(DMS): mitochondrial dysfunction and impaired autophagy in U251 cell line
Source: Cell Death Discov. 2026 Jan 9;12:79. doi: 10.1038/s41420-025-02918-7 (PMC12877171; doi:10.1038/s41420-025-02918-7)

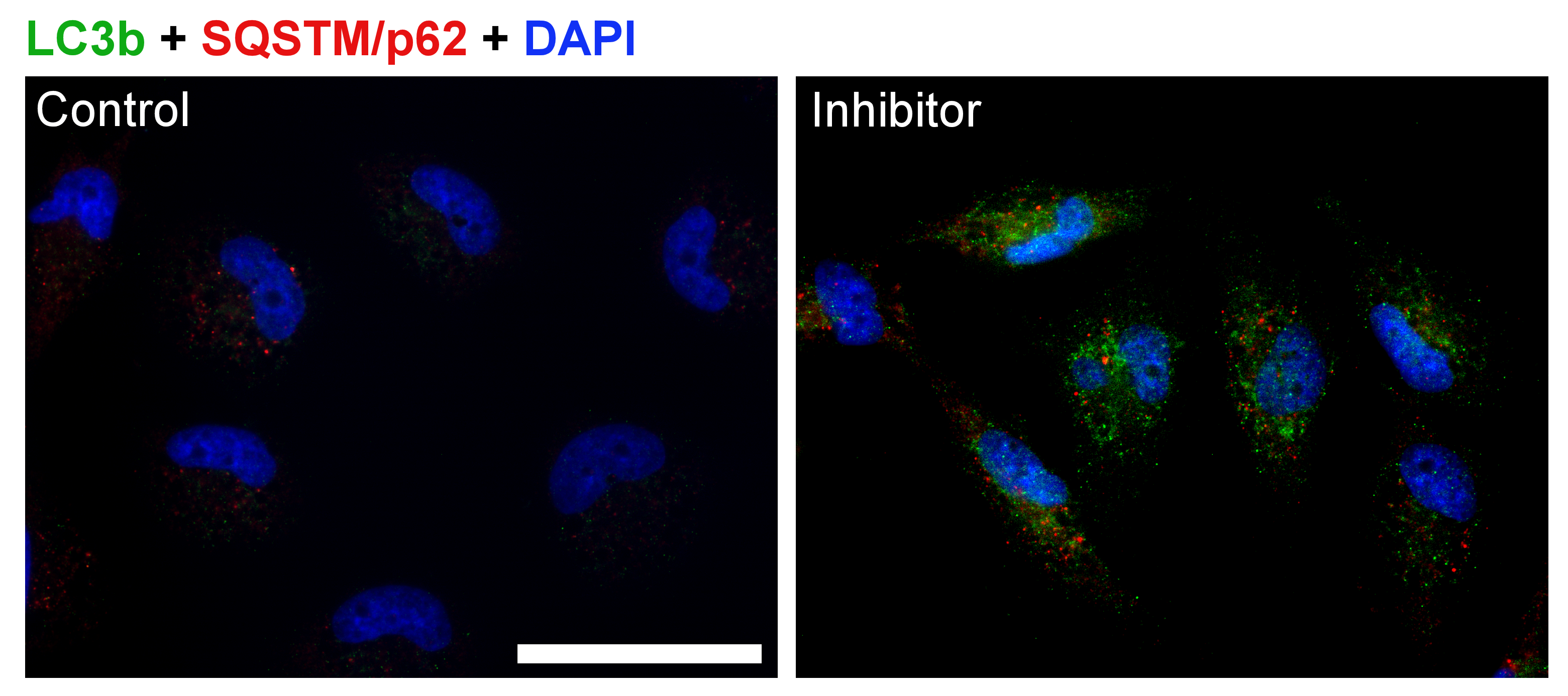

Supplement: Supplementary file 2 — Figure S1 [file 41420_2025_2918_MOESM2_ESM.tif]
